# Mechanical Properties of Cocoon Silk Derivatives for Biomedical Application: A Systematic Review

**DOI:** 10.3390/biomimetics9110675

**Published:** 2024-11-06

**Authors:** Alynah J. Adams, Maria J. Escobar-Domingo, Jose Foppiani, Agustin N. Posso, Dorien I. Schonebaum, Noelle Garbaccio, Jade E. Smith, Lacey Foster, Audrey K. Mustoe, Micaela Tobin, Bernard T. Lee, Samuel J. Lin

**Affiliations:** 1 School of Medicine, Medical College of Wisconsin, Milwaukee, WI 53226, USA; ajadams@mcw.edu; 2 Division of Plastic and Reconstructive Surgery, Beth Israel Deaconess Medical Center, Harvard Medical School, Boston, MA 02215, USA; josefoppiani@yahoo.co.za (J.F.); aposso@bidmc.harvard.edu (A.N.P.); amustoe@bidmc.harvard.edu (A.K.M.); mtobin2@bidmc.harvard.edu (M.T.); blee3@bidmc.harvard.edu (B.T.L.); 3 Department of Plastic and Reconstructive Surgery, University of Minnesota, Minneapolis, MN 55401, USA; 4 Leiden University Medical Centre, Leiden University, 2311 EZ Leiden, The Netherlands; dschoneb@bidmc.harvard.edu; 5 Chobanian & Avedisian School of Medicine, Boston University, Boston, MA 02214, USA; ngarbacc@bidmc.harvard.edu; 6 Northwestern Feinberg School of Medicine, Northwestern University, Chicago, IL 60611, USA; jsmith70@bidmc.harvard.edu; 7 Keck School of Medicine, University of Southern California, Los Angeles, CA 90089, USA; ldfoster@usc.edu

**Keywords:** systematic review, silk, cocoon silk, mechanical properties, biomedicine

## Abstract

Background: Despite cocoon silk’s well-known strength, biocompatibility, and hypoallergenic properties, its potential medical applications remain largely unexplored. This review, therefore, is of significance as it evaluates the mechanical properties and clinical potential of cocoon silk, a material with promising applications in biomaterials and tissue engineering. Methods: We conducted a comprehensive systematic review adhering to PRISMA guidelines. Our focus was on the primary outcomes of tensile strength and elongation at break, and the secondary outcomes included other mechanical properties, applications, and complications. Results: Out of the 192 silk-related studies, 9 met the criteria. These studies revealed that cocoon silk derivatives exhibit a wide range of tensile strength, from 0.464 to 483.9 MPa (with a median of 4.27 MPa), and elongation at break, from 2.56% to 946.5% (with a median of 60.0%). Biomedical applications of cocoon silk derivatives span from tissue regeneration (*n* = 6) to energy harvesting (*n* = 4). Complications often arose from material fragility in non-optimized derivative components. Conclusions: While cocoon silk shows expansive promise due to its suitable mechanical properties and low complication risk, plenty remains to be discovered. Future research is crucial to fully realizing its vast surgical and biomedical potential.

## 1. Introduction

Cocoon silk, predominantly from the domesticated silkworm *Bombyx mori*, has been used clinically for over 4000 years, primarily as suture material [1]. Silk, a natural protein fiber, has garnered significant attention in biomedical research due to its remarkable properties, yielding over 1860 total publications and 1509 within the last ten years. It is biocompatible, hypoallergenic, biodegradable, and readily available in large quantities [2,3]. Cocoon silk is processed by removing sericin, the outer layer, to extract fibroin, the inner core, in a process called degumming, which can then be utilized in various biomedical applications, including wound dressings, tissue scaffolds, and drug-delivery systems [4,5]. The cocoon silk’s fibroin core is favored for its affordability, ease of processing, and established safety profile [2,6].

Cocoon silk fibroin, with its unique advantages like cost-effectiveness, sustainability, and accessibility for large-scale production, is poised to play a significant role in future medical advancements [7,8]. The advances in silk biomaterials have already led to innovative applications, such as resorbable bone screws, vascular grafts, and both wearable and implanted biomedical devices [2,9,10]. However, the journey has had significant progress and innovation. Solutions in maintaining cost-efficient, large-scale production and fully understanding silk-based materials’ long-term biocompatibility and biodegradability, especially in human patients, present exciting opportunities for future research. Over the recent decades, substantial progress has been made in understanding the extensive future implications of silk in biomedical research, clinical management, and mass production.

The development of novel and innovative commercial products is critical for medical advancement and improved clinical outcomes. Cocoon silk is a naturally occurring product produced in multiple layers to protect the silkworm from various environments, predators, and pathogens while promoting thermoregulation and control of the humidity [11]. Cocoon silk from the *Bombyx mori* silkworm is the most widely researched form of domesticated silk product. The investigations yield proof that silk consists of numerous antimicrobial proteins, specifically antibacterial properties against *Staphylococcus aureus* and *Escherichia coli*, although 40 protease inhibitors have been identified from the cocoon of the silkworm, proving inhibition against trypsin and other fungal proteins [11,12].

Silk from the *Bombyx mori* silkworm has a tensile strength ranging from 300 to 740 MPa, comparable to that of high-performance materials like Kevlar, while maintaining an elasticity of approximately 15% before breaking [13]. These characteristics make it ideal for a range of biomedical applications, from sutures to tissue-engineering scaffolds. However, inconsistencies in reporting these mechanical properties across studies limit the comparability and reproducibility of findings. For example, the toughness of silk—a critical property in biomedical applications—has been reported as high as 165 MJ/m^3^, but this value can vary significantly depending on how the silk is processed [13].

Given the diverse biomedical applications of cocoon silk, it is crucial to continue researching and understanding the unique mechanical properties and the processing method of each derivative of cocoon silk. We suspect that the variation in processes for producing silk derivatives stems from the relatively recent discovery of techniques to create these forms from cocoon silk. Due to the novelty of this process, ongoing trial and error is likely hindering the standardization of production methods. However, with time and comprehensive analysis, we are well positioned to refine these variables and optimize the mechanical properties of silk derivatives, ultimately contributing to reproducibility and standardization in future research. These derivatives exhibit variations in tensile strength, elasticity, and toughness, which are essential for optimizing their use in clinical settings. The methods used to quantify and report these properties often differ across studies, making it difficult to draw definitive conclusions. A standardized approach to reporting these properties would significantly enhance the consistency and clarity of cocoon silk research.

To advance the field, it is essential to systematically document and analyze the exact mechanical and biochemical properties reported in each study. Standardizing a reporting form that includes tensile strength, elasticity, toughness, molecular weight and antimicrobial activity would enhance the consistency and clarity of research in this field and pave the way for a more comprehensive understanding of cocoon silk’s full potential. By adopting this approach, future research can more accurately assess cocoon silk’s full potential and guide its development into a reliable, standardized biomaterial for clinical use. This standardization is critical not only for improving the reproducibility of studies but also for facilitating the translation of research findings into commercial medical products that offer improved safety and efficacy for patients.

## 2. Materials and Methods

### 2.1. Eligibility Criteria

Eligibility criteria included studies of cocoon silk derivatives for medical applications that included our primary and secondary outcomes, observational studies, and clinical trials published in English, French, or Spanish. Editorials, literature reviews, meta-analyses, commentary reports, abstracts, and letters to the editors were excluded from this review because sources like these typically do not present original research data. Due to this lack of originality, the methodological rigor required for inclusion was unachievable. In addition, studies reporting treatments involving materials other than cocoon silk, articles without mechanical properties, and ongoing and cadaveric studies were excluded. Ongoing studies are not able to provide convincing final results, and cadaveric studies have inherent limitations in translating findings to living subjects. The full eligibility criteria are accessible at PROSPERO.

### 2.2. Information Sources

**Figure 1:**
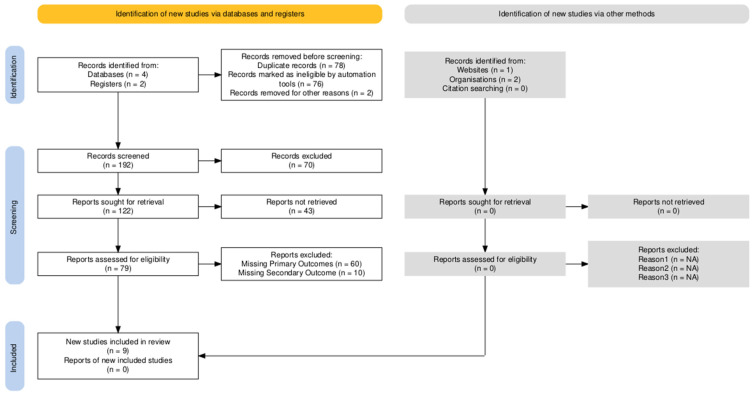
PRISMA flow diagram [14,15].

A comprehensive systematic literature review was performed in June 2024 following the Preferred Reporting Items for Systematic Reviews and Meta-Analysis (PRISMA) guidelines (Figure 1) [14,15]. The databases searched included MEDLINE, Web of Science, and Cochrane Central Register.

### 2.3. Search Strategy

The search strategy was designed by an experienced librarian using terms, subject headings, and keywords related to cocoon silk, biomaterial, bioengineering, biology, clinical outcomes, mechanical properties, physical properties, biomedical application, sericin, fibroin, wound dressing, tissue scaffolds, drug delivery, cost-effectiveness, biocompatibility, biodegradability, medical therapy, and medical application. A detailed overview of the search strategy can be found in Table 1.

### 2.4. Selection Process

The online systematic review program, Covidence, was used to upload the search results [16]. A two-stage screening process was conducted by two independent reviewers (A.A. and A.P.) for study selection. First, subjects were reviewed by screening for article titles and abstracts. Next, a full-text analysis took place by the same two reviewers. All discordances were resolved by a third researcher (M.J.E. or J.F.) moderated by discussion until a joint decision was made. The protocol for this systematic review was registered on PROSPERO under the identification number CRD42024557379 [17].

### 2.5. Data Extraction

Using predesignated variables, six independent reviewers (A.A, A.P, D.S, N.G, L.F., and J.S) extracted the data from the final articles. These variables included the first author’s last name, publication year, journal title, country or region of study, type of study (in vivo, in vitro, or in situ), the model organism or synthetic material utilized, the production method, the component of the cocoon silk fiber, and the primary and variable secondary outcomes.

### 2.6. Data Items

The primary outcomes sought were tensile strength and elongation at break. Our secondary outcomes included other recorded mechanical properties, clinical applications, and complications. Other variables for which we sought data included author, title, year, publication year, journal, country of publication, and experimental type of approach.

### 2.7. Statistical Analysis

The studies included in this systematic review varied considerably and barred the possibility of performing any analysis beyond a qualitative synthesis.

### 2.8. NIH Quality Assessment

In order to evaluate the potential for bias, the quality assessment tool developed by the National Institute of Health (NIH) was employed. Each study was classified according to its quality level as either “poor”, “fair”, or “good” (Table S1).

## 3. Results

### 3.1. Study Selection

Out of 270 records identified, 192 abstracts and titles were screened after the removal of duplicates. Following this, 122 full-text articles were sought for retrieval. Ultimately, nine studies met the inclusion criteria for this systematic review (Table 2). These nine studies were selected for inclusion due to their strict compliance with our predefined inclusion criteria, as outlined during the screening process according to PRISMA guidelines. They specifically examined cocoon silk derivatives, exploring their mechanical properties, biomedical applications, and any associated complications.

**Table 1:** Search strategy and database sources.

Database	Search
PubMed/ MEDLINE (AB = 111)	(“cocoon silk” [Title/Abstract] OR “silk fibroin” [Title/Abstract] OR “sericin” [Title/Abstract] OR “silkworm silk” [Title/Abstract] OR “Bombyx mori silk” [Title/Abstract] OR “spider silk” [Title/Abstract] OR “spider dragline silk” [Title/Abstract] OR “spidroin” [Title/Abstract]) AND (“medical application” [Title/Abstract] OR “surgical application” [Title/Abstract] OR “biomedical application” [Title/Abstract] OR “medical devices” [Title/Abstract] OR “surgical devices” [Title/Abstract] OR “biomedical devices” [Title/Abstract] OR “translational science” [Title/Abstract] OR “translational medicine” [Title/Abstract])
Web of Science (AB = 139)	(AB = (“cocoon silk” OR “silk fibroin” OR “sericin” OR “silkworm silk” OR “Bombyx mori silk” OR “spider silk” OR “spider dragline silk” OR “spidroin”)) AND AB = (“medical application” OR “surgical application” OR “biomedical application” OR “medical devices” OR “surgical devices” OR “biomedical devices” OR “translational science” OR “translational medicine”)
Web of Science (TI = 14)	(TI = (“cocoon silk” OR “silk fibroin” OR “sericin” OR “silkworm silk” OR “Bombyx mori silk” OR “spider silk” OR “spider dragline silk” OR “spidroin”)) AND TI = (“medical application” OR “surgical application” OR “biomedical application” OR “medical devices” OR “surgical devices” OR “biomedical devices” OR “translational science” OR “translational medicine”)
Cochrane Central Register (AB + TI + AK = 0)	(“cocoon silk” OR “silk fibroin” OR “sericin” OR “silkworm silk” OR “Bombyx mori silk” OR “spider silk” OR “spider dragline silk” OR “spidroin”) AND (“medical application” OR “surgical application” OR “biomedical application” OR “medical devices” OR “surgical devices” OR “biomedical devices” OR “translational science” OR “translational medicine”)

### 3.2. Studies Characteristics

The selected studies were conducted between 2007 and 2024 and were distributed across various regions, including the USA (11%), Europe (22%), and Asia (56%), with one study (11%) from another region [18,19,20,21,22,23,24,25,26]. The experimental approaches employed in these studies varied, with 22% using in situ methods, 44% using in vitro methods, 33% employing a mix of in vivo and in vitro methods, and none using in vivo methods exclusively [18,19,20,21,22,23,24,25,26]. Different silk development protocols were utilized, including additive manufacturing, cross-linking, electrospinning, freeze-drying with compression, Schiff’s base reaction, and solution casting methods (Table 3) [18,19,20,21,22,23,24,25,26]. 

**Table 2:** Silk material products by publication, author, and study type.

Title of Publication	First Author, Country	Year	Study Type	Product
Polyvinylidene Fluoride/Silk Fibroin-Based Bio-Piezoelectric Nanofibrous Scaffolds for Biomedical Application [24].	Chen, K.; Korea	2022	In vitro	Polyvinylidene flouride (PVDF/SF)
Preparation and Characterization of a Silk Fibroin/Polyurethane Fiber Blend Membrane Containing Actinomycin X2 with Excellent Mechanical Properties and Enhanced Antibacterial Activities [23].	Tariq, Z.; China	2023	In vitro	Polyurethane fiber (PUF) and silk-fibroin-containing sctinomycin X2 (Ac.X2)
Cross-Linking of Dialdehyde Carboxymethyl Cellulose with Silk Sericin to Reinforce Sericin Film for Potential Biomedical Application [19].	Wang P.; China	2019	In vitro	Dialdehyde carboxymethyl cellulose (DCMC)
Biocompatible Silk/Polymer Energy Harvesters Using Stretched Poly (Vinylidene Fluoride-co-Hexafluoropropylene) (PVDF-HFP) Nanofibers [22].	Najjar, R.; USA	2017	In vitro	Polyvinylidene flouride Hexafluropropylene nanofibers with glycerol
Superb Silk Hydrogels with High Adaptability, Bioactivity, and Versatility Enabled by Photo-Cross-Linking [21].	Huang, R.; China	2024	In vitro, in vivo	Riboflavin and H_2_O_2_
Modification of Sericin-Free Silk Fibers for Ligament Tissue Engineering Application [18].	Liu, H.; Singapore	2007	In vitro, in vivo	SF replaced by gelatin
A Multi-Layered Nerve Guidance Conduit Design Adapted to Facilitate Surgical Implantation [25].	Belanger, K.; France	2018	In vitro, in vivo	Tri-layered nanofiber
Characterization of Direct Ink Write Pure Silk Fibroin Based on Alcohol Post-Treatments [26].	Casanova-Batllle, E.; Spain	2022	In situ	Natural SF vs. EtOH SF
Silk and Silk Composite Aerogel-Based Biocompatible Triboelectric Nanogenerators for Efficient Energy Harvesting [20].	Mi, HY.; China	2020	In situ	SF aerogel-based triboelectric nanogenerator (STENG)

**Table 3:** General characteristics of included studies, silk development protocols, and percentage of silk components.

	Total	%
No. of Publications Included	9	100
Year range	2007–2024	
Countries Publications Drawn From (%)		
USA	1	11
Europe	2	22
Asia	5	56
Other	1	11
Experimental Approach (%)		
In situ	2	22
In vitro	4	44
In vivo	0	0
In vivo and in vitro mix	3	33
Silk Development Protocol (%)		
Additive manufacturing method	1	11
Cross-linking	2	22
Electrospinning	3	33
Freeze-drying with compression	1	11
Schiff’s base reaction	1	11
Solution casting method	1	11
Cocoon Silk Component (%)		
Fibroin	8	89
Sericin	1	11
Journal Category (%)		
Tissue engineering	1	11
Chemistry	5	56
Medicine and health	1	11
Nanoscience	1	11
Biomedical materials	1	11

### 3.3. Mechanical Properties

The primary outcomes of tensile strength and elongation at break were reported across all studies. The tensile strength of cocoon silk derivatives ranged from 0.70 to 483.9 MPa, with a median tensile strength of 4.27 MPa [18,19,20,21,22,23,24,25,26]. The elongation at break varied from 2.56% to 946.5%, with a median of 60.0% [18,19,20,21,22,23,24,25,26]. Secondary outcomes, including Young’s compressive modulus, surface descriptors, fiber diameter, piezoelectric voltage, crystallinity, FT-IR, porosity, pore size, and swelling ratio, were reported in varying frequencies (Table 4).

**Table 4:** Cocoon silk composition, biomedical applications, and complications.

Cocoon-Silk-Derivative Composition	*n*	%
Cross-linked gelatin replacement for sericin	1	11
Dialdehyde carboxymethyl cellulose (DCMC)	1	11
Silk-fibroin-aerogel-based triboelectric nanogenerator	1	11
Natural silk fibroin vs. EtOH-treated silk fibroin	1	11
Polyvinylidene fluoride (PVDF)	1	11
Polyvinylidene fluoride (PVDF) and hexafluoropropylene (HFP)	1	11
Polyurethane fiber (PUF) and actinomycin X2 (Ac.X2)	1	11
Riboflavin and H_2_O_2_	1	11
Tri-layered nanofibers	1	11
Biomedical Applications *	*n*	%
Number of studies with biomedical applications reported	9	100
Cell proliferation and tissue engineering	6	30
Energy-harvesting devices	4	20
Antimicrobial hydrogels	3	15
Orthopedic screws	1	5
Microneedles and microcarriers	2	10
Drug delivery	2	10
Nerve guidance conduit	1	5
Cardiovascular stents	1	5
Complications	*n*	%
Number of studies with reported complications	7	78
Fragility	4	57.1
Cytotoxicity	1	14.3
Sensitivity to temperature and humidity	1	14.3
Fast degradation	1	14.3
Rare inflammatory reaction	1	14.3
Mechanical Properties of Cocoon Silk Derivatives *	*n*	%
Primary Outcomes:	9	100
Tensile strength		
Elongation at break		
Secondary Outcomes:		
Number of studies with secondary outcomes reported	98	100
Young’s compressive modulus (GPa)	8	89
Surface descriptors	8	89
Fiber diameter (mm)	2	89
Piezoelective (V)	2	22
Crystallinity (%)	8	22
FT-IR (cm^−1^)	2	89
Porosity (%)	2	22
Pore size (nm)	5	22
Swelling ratio (%)		56

**Figure 2:**
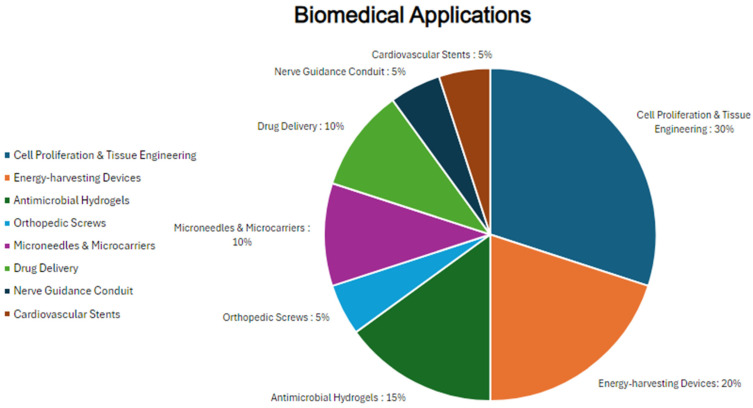
Biomedical applications of included studies.

* Included as Figure 2.

### 3.4. Biomedical Applications of Cocoon Silk Derivatives

Cocoon silk derivatives were applied in various biomedical fields. The applications included tissue engineering and cell proliferation (30%), energy-harvesting devices (20%), antimicrobial hydrogels (15%), orthopedic screws (5%), microneedles and microcarriers (10%), drug-delivery systems (10%), nerve guidance conduits (5%), and cardiovascular stents (5%) (Figure 2) [18,19,20,21,22,23,24,25,26]. Acquiring detailed information about these applications is highly beneficial to the clinical and medical community because it helps identify novel materials and methods that can improve patient outcomes, enhance the durability and effectiveness of medical devices, and provide new solutions for complex medical conditions.

### 3.5. Complications

Complications were reported in seven of the nine studies included (100%). The most common complication reported in the studies was fragility (57.1%) [21,24,25,26], followed by cytotoxicity (14.3%) [23], sensitivity to temperature and humidity during storage (14.3%) [18], fast degradation time (14.3%) [26], and rare inflammatory reactions (14.3%) [18] (Table 4).

### 3.6. Analysis of Individual Studies

#### 3.6.1. Cross-Linked Gelatin Replacement for Sericin

Liu H. et al. (2007) conducted both in vitro and in vivo studies to enhance the properties of sericin-free silk fibers by replacing sericin with cross-linked gelatin for ligament tissue-engineering applications. The synthetic product was prepared by immersing the sericin-free silk fibers in various concentrations of aqueous gelatin solution. The tensile strength for the 5% gelatin-modified silk fibers was reported at 483.85 MPa, and the elongation at break was 21%. The secondary properties, which include Young’s compressive modulus (10,500 MPa), a smooth surface, fiber diameter (10 µm), porosity (110%), and a swelling ratio (45× higher than native silk fibers) for 5% gelatin-modified fibers, were the strongest variation studied (Table 2). The study demonstrated improved mechanical properties and biocompatibility of the modified silk fibers, closely resembling native silk fibers. However, complications included variations in swelling properties, with the 5% gelatin-modified fibers exhibiting significantly higher swelling compared to native silk fibers, leading to potential issues in maintaining consistent mechanical properties. The potential for cytotoxicity and immune response, however faint, remained a concern, although the in vivo studies showed minimal inflammatory response, indicating good biocompatibility. The use of modified silk fibers for ligament tissue engineering has significant implications, as it can lead to improved methods for repairing ligament injuries, which are common and often debilitating. The study concluded that the modified silk fibers with gelatin could serve as a promising scaffold for ligament tissue engineering, provided that the swelling and mechanical stability are carefully managed [18].

#### 3.6.2. Dialdehyde Carboxymethyl Cellulose (DCMC)

Wang P. et al. (2019) conducted an in vitro study that investigated the reinforcement of sericin film using dialdehyde carboxymethyl cellulose (DCMC) derived from periodate oxidation of carboxy-methyl cellulose. The synthetic product was prepared via Schiff’s base reaction, using cocoon sericin. The tensile strength for the D12 composite was reported at 41.2 MPa with an SD of ±1.11 MPa, and the elongation at break was 39%. Additionally, the secondary mechanical properties include Young’s compressive modulus (1200 MPa), confirmatory FT-IR band (3286 cm^−1^), and the swelling ratio (130%) (Table 2). The study demonstrated that the DCMC/sericin film exhibited excellent blood compatibility, cytocompatibility, and cell proliferation activity, making it suitable for biomedical applications such as wound dressing, artificial skin, and tissue engineering. However, complications arose due to the sensitivity of the films to temperature and humidity, which could significantly affect their mechanical properties, including hydrophilicity, tensile strength, and elongation at break. The cross-linking process itself could introduce variability in the material properties, potentially leading to inconsistent performance in biomedical applications. Overall, these films have potential uses in creating more durable and flexible biomedical devices, which are crucial for various medical applications, including wound healing and tissue scaffolding [19].

#### 3.6.3. Silk-Fibroin-Aerogel-Based Triboelectric Nanogenerator

Mi, Hao-Yan et al. (2020) conducted an in situ study to develop a silk fibroin aerogel-based triboelectric nanogenerator (STENG) aimed at efficient energy harvesting. The synthetic product was prepared using freeze-drying and compression methods, utilizing cocoon silk fibroins. The tensile strength of the silk aerogel was reported at 0.25 MPa, while the cellulose nanofiber (CNF) aerogel showed a higher tensile strength of 0.7 MPa. The elongation at break varied significantly with the concentration of the silk aerogel, being brittle at around 1% for 1% silk aerogel, and increasing to over 3% for 2% silk aerogel and over 10% for 4% silk aerogel. The study highlighted the unique rough nanoporous structure of the aerogels, with raw silk fibers having a diameter of approximately 6 μm. The STENG exhibited a piezoelectric voltage of 52.8 V, a short circuit current of 5.2 μA, and a power density of 0.37 W/m^2^ on a 1 MΩ external resistor. The CNF alone had a porosity of 88.5%, while the CNF/silk composite had a slightly lower porosity of 73.3% (Table 2) [19]. The research demonstrated the potential of STENGs for implantable energy-harvesting devices due to their high performance and biocompatibility. However, the study also noted some challenges, including the brittleness of low-concentration silk aerogels and the necessity for precise control of aerogel composition to maintain optimal mechanical properties and biocompatibility. This innovative approach suggests that triboelectric nanogenerators could be effectively used in the development of novel medical devices and wearable electronics, leveraging the unique properties of silk fibroin aerogels to create efficient and biocompatible energy-harvesting solutions [20].

#### 3.6.4. Riboflavin and H_2_O_2_

Huang R. et al. (2024) conducted an in vitro and in vivo study to develop silk-based hydrogels with enhanced mechanical and biological properties. The synthetic product was prepared using a sustainable cyclic photo-cross-linking reaction involving riboflavin (RF) and hydrogen peroxide (H_2_O_2_), with cocoon fibroin as the base material. The tensile strength of the hydrogel was reported at 12.5 MPa at the first compression cycle, 10.5 MPa after 500 cycles, and 8.0 MPa after 1000 cycles. The elongation at break was over 60%. The secondary mechanical properties included Young’s compressive modulus (~3.5 MPa), a smooth surface, fiber diameter (~400 µm), crystallinity peak (~24.3°), FT-IR (1535–1514 cm^−1^), and swelling properties (390%) (Table 2). The study highlighted the hydrogel’s resilience and complete recovery after each compression cycle, demonstrating its potential for various biomedical applications, including bone screws and cell-delivery platforms. However, complications included the fragility of the hydrogels and the challenges in balancing superior mechanical properties with efficient preparation, as increasing reactants led to more cross-linking, reducing pore size and swelling ratio. The process of photo-cross-linking, while beneficial for enhancing mechanical properties and bioactivity, could be time-consuming, affecting the practicality of these hydrogels in urgent medical situations. Additionally, the sensitivity of the hydrogels to environmental factors such as light and temperature could complicate their storage and handling. These hydrogels could be used for wound dressings and tissue regeneration, offering enhanced healing properties and adaptability to various medical needs, including drug delivery, disease diagnosing, microneedling, microcarriers, and bone screw development [21].

#### 3.6.5. Polyvinylidene Fluoride (PVDF) and Hexafluoropropylene (HFP)

Najjar R. et al. (2017) conducted an in vitro study to develop energy-harvesting devices using a composite of PVDF-HFP nanofibers and glycerol-enhanced silk fibroin (SF). The synthetic product was prepared using electrospinning and stretching methods, utilizing cocoon fibroin. The tensile strength for the SF–glycerol composite was reported at 2.67 MPa, with an ultimate elongation at break of 117.75%. Secondary mechanical properties included Young’s compression modulus (1.162 MPa), a surface description (smooth), fiber diameter (electrospun nanofibers: 600–1600 nm; stretched nanofibers: 300–700 nm), piezoelectric volts (2.6 V), and a confirmatory FT-IR (1595–1705 cm^−1^) (Table 2). The study demonstrated that the SF–glycerol composite exhibited higher strength and larger ultimate strain compared to natural silk fibroin, making it a promising candidate for biomedical applications such as wearable health monitoring sensor systems and energy-harvesting devices. However, complications were noted in achieving the precise concentration of each material in the composite and cytotoxicity, as incorrect ratios could lead to phase separation and fragility or ineffective mechanical properties. This technology could significantly benefit wearable and implantable devices, like pacemakers and automatic implantable cardioverter defibrillators (AICDs) by providing a continuous power supply through energy harvesting [22].

#### 3.6.6. Polyurethane Fiber (PUF) and Actinomycin X2 (Ac.X2)

Tariq Z. et al. (2023) conducted an in vitro study to develop a flexible and antibacterial blend membrane using silk fibroin (SF) and polyurethane fiber (PUF)-containing actinomycin X2 (Ac.X2). The synthetic product was prepared using a green chemical method/solvent casting method, with cocoon silk fibroin as the type of cocoon silk. The tensile strength for the 50% SF/PUF composite was reported at 25.7 MPa, and the elongation at break was 946.5% ± 38.06%. The secondary mechanical properties of Young’s compressive modulus (0.2 MPa), fiber diameter (35 mm), and FT-IR (3288 cm^–1^) (Table 2). The study highlighted that while the blend membranes demonstrated good mechanical properties and antibacterial activities, complications arose due to the potential cytotoxicity of actinomycin X2, requiring the precise control of product percentages to balance cytotoxicity and antimicrobial properties effectively. This combination makes the blend suitable for a range of biomedical applications, specifically providing a new avenue for developing hygienic wound dressings. This blending process also has the potential to introduce inconsistencies in fiber properties, leading to potential issues in reproducibility and reliability. Such fibers could be used in flexible biomedical devices, offering improved performance and adaptability in various clinical scenarios [23].

#### 3.6.7. Polyvinylidene Fluoride (PVDF)

Chen K. et al. (2022) conducted an in vitro study that explored the use of polyvinylidene fluoride (PVDF) mixed with silk fibroin (SF) to create a composite material with potential biomedical applications. The synthetic product was prepared using the electrospinning method, with cocoon fibroin as the type of cocoon silk. The tensile strength for the PVDF/SF 20% composite was reported at 2.62 MPa, and the elongation at break was 52% (Table 2). The study revealed that while the addition of SF enhanced the tensile strength and biocompatibility, leading to improved cell proliferation, complications arose due to the mat’s low porosity and fragility. Specifically, the densification of the mat with small pores impeded cell penetration, thus presenting a significant challenge for further application in bio-piezoelectric devices. The secondary properties included a surface description (dense fibrous structure), piezoelectric volts (“rather low”), crystallinity (20%), and a confirmatory FT-IR (1275–840 cm^−1^) [24]. In this study, complications included the fragility of the scaffolds and their sensitivity to environmental conditions. With an appropriate calibration of SF, there were obvious improvements in tensile strength, enhanced cell proliferation rates, and sufficient voltage generated to be useful in piezoelectric fields, but the standardization of mechanical property value reporting would help when comparing silk-derived products for reproduction. These scaffolds could be used in tissue engineering to promote cell growth and tissue regeneration, providing innovative solutions for repairing damaged tissues.

#### 3.6.8. Tri-Layered Nanofibers

Belanger K. et al. (2018) conducted an extensive study both in vitro and in vivo to create an advanced nerve guidance conduit using silk fibroin via electrospinning. This study aimed to address the complexities of nerve regeneration by utilizing a sophisticated multi-layered design. The synthetic product was tested in vivo on Sprague Dawley rats. The conduit was constructed from a tri-layered nanofiber material, rolled into a micro-channeled conduit, and further enveloped by a jacket layer of the same tri-layered material. The electrospinning method was used to achieve this intricate structure, with cocoon fibroin as the base material. The tensile strength of the aligned fibers was reported at 2.90 MPa, while the tri-layered fibers showed a slightly lower tensile strength of 2.6 MPa. Randomly aligned fibers had the lowest tensile strength at 0.5 MPa. The aligned fibers demonstrated the highest resistance to tearing, particularly when not twisted. The elongation at break was 85% for the aligned fibers, 263% for the tri-layered configuration, and 250% for the randomly aligned fibers. In terms of Young’s compressive modulus, the aligned fibers were the most resistant at 14.9 MPa, followed by the tri-layered fibers at 13.0 MPa and the randomly aligned fibers at 1.2 MPa. The fiber diameter (200–600 nm), piezoelectric voltage (0.134 V), and the crystallinity of the silk I structure (24.3 degrees) can be observed in Table 2. Despite these promising results, the study identified several complications. The fragility of the conduit posed challenges during surgical implantation, as blunt edges could deform nerve fascicles, making precise micro-suturing difficult. Additionally, excessive tensile stress on large nerve segments necessitated the use of grafts rather than direct suturing to avoid further damage. The research concluded that the multi-layered nerve guidance conduit, with its unique tri-layered design and enhanced mechanical properties, holds significant potential for nerve repair applications. The study emphasized the need for careful handling and precise implementation to maximize the conduit’s benefits while mitigating the identified complications. This innovative approach offers a promising pathway for improving nerve regeneration outcomes in clinical settings [25].

#### 3.6.9. Natural Silk Fibroin vs. EtOH-Treated Silk Fibroin

Finally, Casanova-Batlle, Enric et al. (2022) conducted an in situ study to evaluate the mechanical properties of silk fibroin (SF) processed using a novel additive manufacturing method for medical device customization. This study compared the untreated SF (no tx) with ethanol-treated SF (EtOH). The synthetic product was prepared using an additive manufacturing method with cocoon silk fibroin. The tensile strength of the untreated SF was reported at 8.84 ± 3.73 MPa, while the EtOH-treated SF showed a reduced tensile strength of 4.27 ± 2.68 MPa. The elongation at break for untreated SF was 5.87 ± 2.81% compared to 2.56 ± 0.96% for the EtOH-treated SF. Young’s compressive modulus varied significantly, with untreated SF at 637 ± 300 MPa and EtOH-treated SF at 293 ± 151 MPa. Secondary mechanical properties include a surface description (flat), fiber diameter (1.6 mm), FT-IR (1230, 1261, and 1696 cm^–1^), and swelling properties (56.69–60.06%) (Table 2). The study highlighted several complications associated with the alcohol post-treatment of SF. The ethanol treatment led to increased fragility and brittleness, faster degradation, and lower mechanical properties compared to untreated SF. The research emphasized that molecular weight (MW) is a critical parameter affecting the degradation rate and mechanical properties of silk fibroin. Longer degumming steps or higher concentrations of Na_2_CO_3_ reduced the MW, impacting the SF’s performance [26]. Despite these challenges, the study demonstrated the potential of using the DIW technique to print highly concentrated SF ink for customizing medical devices, such as cardiovascular stents and drug-delivery systems. The research found that silk fibroin processed with alcohols became brittle, which is necessary for achieving flexible regenerated SF films. The study also noted an increase in elasticity modulus and ultimate tensile strength (UTS) with alcohol treatment, but prolonged exposure to alcohols decreased these properties compared to untreated fibroin. The findings suggest that while alcohol-treated silk fibroin shows promise for specific biomedical applications, careful consideration of processing conditions is essential to optimize the mechanical properties and ensure the material’s suitability for medical device applications.

## 4. Discussion

The systematic review of cocoon silk derivatives has revealed significant insights into their mechanical properties, biomedical applications, and associated complications. This discussion synthesizes the findings from the studies included, highlighting the implications for future research and clinical practice.

### 4.1. Tensile Strength and Elongation at Break as Mechanical Properties

In evaluating the mechanical properties to prioritize for comparison in this article, tensile strength and elongation at break emerged as the most frequently documented metrics. Tensile strength, defined as the force per unit area required to break a material, is a critical parameter for assessing the mechanical performance of polymeric materials [27]. Without tensile strength testing, distinguishing the mechanical characteristics of new cocoon silk derivatives would be difficult, underscoring its importance for material comparison and innovation. Similarly, elongation at break is invaluable for evaluating the performance of newly produced silk fibers. This parameter, which describes the rupture behavior of a material, is an indirect measure of the evolution of chain scission [28,29]. Together, tensile strength and elongation at break offer a comprehensive view of the biomechanical properties, allowing researchers to better understand the performance and potential of the materials being used.

### 4.2. The Mechanical Properties of Natural Cocoon Silk

Natural *Bombyx mori* cocoon silk is remarkably strong, with a recorded tensile strength of 500 MPa [30,31]. After degumming—removing the sericin outer layer—the ultimate tensile strength of silk fibroin increases, ranging from 610 to 690 MPa [30]. Additionally, tests on cocoon silk revealed an impressive elongation at break of 15%. It has been suggested that the variability in the strength and elasticity of individual silk strands is largely influenced by the speed at which *Bombyx mori* spins its silk [30].

The tensile strength of cocoon silk derivatives varied widely across the studies, with a range from 0.70 to 483.9 MPa [18,20]. The elongation at break also exhibited substantial variability, from ~1% to 946.5% [18,23]. These results underscore the material’s versatility and adaptability for different biomedical applications. Notably, the modification of sericin-free silk fibers with gelatin, as reported by Liu H et al., significantly enhanced their mechanical properties, achieving a maximum tensile stress of 483.85 MPa and a modulus of 10.50 GPa [18]. These enhancements appear to be superior for applications requiring high strength and durability, such as ligament-tissue-engineering and orthopedic devices. The study documented a singular complication, albeit vaguely described, involving a rare and atypical inflammatory reaction.

### 4.3. Biomechanical Applications of Cocoon Silk Derivatives

The biomedical applications of cocoon silk derivatives are diverse, spanning tissue engineering, energy harvesting, antimicrobial hydrogels, orthopedic screws, microneedles, drug-delivery systems, nerve guidance conduits, and cardiovascular stents. These applications highlight the material’s potential to address various clinical needs. For instance, the use of silk fibers modified with gelatin for ligament tissue engineering offers a promising solution for repairing ligament injuries, which are common and often result in significant joint dysfunction [18]. Similarly, the development of silk composite aerogels for energy harvesting could revolutionize the powering of implanted medical devices, reducing the need for external power sources or frequent battery replacements [20].

### 4.4. Complications and Limitations of Cocoon Silk Derivatives

Despite the promising applications, several complications were identified across the studies, each of which poses significant challenges to these materials’ practical use and effectiveness. The complications found in each of these studies underscore the substantial and occasionally vague limitations in the production of these novel cocoon silk derivatives.

#### 4.4.1. Fragility

Fragility was common, especially in aerogels and fibers modified for energy harvesting. For instance, silk fibroin aerogels designed for triboelectric nanogenerators exhibited brittleness at low concentrations, which limits their mechanical stability and makes them less suitable for dynamic environments where flexibility and durability are crucial. This fragility could result in device failure when used in wearable or implantable applications, where materials must withstand mechanical stresses over time. Similarly, the fragility of tri-layered nanofiber conduits for nerve regeneration created difficulties during surgical implantation, where even slight deformation can disrupt precise suturing and nerve repair.

#### 4.4.2. Sensitivity to Environmental Factors

Another widespread complication was sensitivity to environmental factors, particularly temperature and humidity. For example, silk sericin films cross-linked with dialdehyde carboxymethyl cellulose demonstrated significant changes in mechanical properties under varying environmental conditions. Such sensitivity could lead to material degradation or failure in long-term applications, especially in biomedical devices where consistent performance is essential. This is particularly concerning for wound dressings and tissue scaffolds, where even minor fluctuations in mechanical properties could impair their ability to support healing processes effectively.

#### 4.4.3. Cytotoxicity

Cytotoxicity was another concern, although less frequent. In some studies, such as the development of PVDF-HFP nanofibers, improper material ratios led to cytotoxic effects, making the composite unsuitable for biomedical applications. For instance, phase separation in these composites could create localized areas of mechanical weakness or excessive rigidity, compromising their biocompatibility and functional performance. Ensuring biocompatibility is especially critical when these materials are intended for implantable devices or tissue engineering, as any adverse biological reaction could negate the intended benefits of the device.

#### 4.4.4. Swelling

Swelling properties also introduced complications. For example, gelatin-modified silk fibers exhibited significantly higher swelling ratios compared to native silk fibers, which could lead to instability in their mechanical properties over time. Excessive swelling can cause alterations in fiber dimensions, reducing the material’s ability to maintain structural integrity in applications such as ligament repair, where mechanical stability is paramount. Additionally, hydrogels enhanced with riboflavin and hydrogen peroxide faced challenges in balancing mechanical strength with swelling, as increased cross-linking improved durability but reduced swelling capacity, potentially limiting their use in applications requiring flexibility and high porosity, like bone scaffolds or drug-delivery platforms.

#### 4.4.5. Variability in Silk Derivatives

Lastly, variability in the production of derivative silk products and composition control led to inconsistencies in performance. This was particularly evident in films prepared with dialdehyde carboxymethyl cellulose, where minor alterations in cross-linking introduced variability in their mechanical and swelling properties. Such inconsistencies could undermine the reliability of these materials in clinical settings, where reproducibility and standardization are crucial for patient safety and treatment efficacy. Additionally, in applications like tissue engineering or energy harvesting, variability in material properties could lead to device malfunction or inconsistent therapeutic outcomes.

In summary, while these advanced silk-based materials offer significant potential for a wide range of biomedical applications, several key challenges must be addressed to ensure their effectiveness and reliability. Fragility, environmental sensitivity, cytotoxicity, and variability in mechanical properties were common complications that could limit their practical use, particularly in dynamic or long-term medical settings. Overcoming these challenges will be essential for fully harnessing the benefits of these materials in applications such as tissue engineering, wound healing, and energy harvesting. Continued design refinement and careful control of their synthesis processes will be crucial to optimize their performance and ensure their successful integration into clinical practice.

### 4.5. Clinical Applications of Cocoon Silk Derivatives

The findings from this review have several implications for clinical practice and future research. The enhanced mechanical properties of modified silk derivatives suggest their potential for developing more durable and effective biomedical devices and materials. For example, gelatin-modified silk fibers’ improved tensile strength and modulus make them suitable for high-stress applications such as ligament and tendon repairs [18]. Additionally, the biocompatibility of these materials, especially cocoon silk fibroin derivative products, as evidenced by minimal inflammatory responses both in vitro and in vivo, supports their potential for widespread clinical use.

However, addressing the identified complications is crucial for advancing the clinical application of cocoon silk derivatives. Future research will likely focus on optimizing these materials’ mechanical properties and environmental stability. Developing standardized protocols for the silk modification and standardized reporting of the mechanical properties will help researchers better compare the silk-derivative materials. With several of our primary and secondary outcomes not being reported, there was an important missed opportunity to truly understand how these materials stand up to each other. Therefore, these studies can potentially advance medical research at an unmitigated pace.

### 4.6. Environmental Impact of Cocoon Silk Production

Along with perfecting these methods of producing derivative silk materials and creating a standardized reporting method, researchers should work toward reducing the environmental and costly impacts of derivative silk manufacturing. Jones J.A. et al. discuss in detail how their aqueous solubilization method for creating recombinant spider silk protein materials is cost-effective, environmentally friendly, and scalable for mass production [32]. The aqueous solubilization method for recombinant spider silk proteins eliminates the need for expensive and hazardous organic solvents. This method achieves nearly 100% protein solubilization and recovery by using water and microwave heating, making it scalable for mass production. This approach is efficient and quick and significantly reduces environmental impact, as it minimizes the use of toxic chemicals and waste generation. Developing such sustainable production methods is crucial for lowering costs and creating eco-friendly materials, which have wide applications in fields like medicine and engineering [32]. With more research, it could be possible to mirror the successful processes of recombinant spider silk to enhance cocoon silk production.

### 4.7. Future Directions Discussion Conclusion

Cocoon silk derivatives exhibit promising mechanical properties and diverse biomedical applications. However, addressing the complications of fragility, environmental sensitivity, and cytotoxicity is essential for their successful clinical implementation. Future research should optimize these materials’ properties and explore new applications to fully realize their potential in the medical field. The ability to reasonably compare these materials using definitive mechanical properties like tensile strength, elongation at break, Young’s compression modulus, and other secondary components will make the goal of innovative silk-based materials a more insightful reality. Yan et. al. agree with standardized reporting of mechanical properties, specifically endorsing the inclusion of tensile strength, elongation at break, Young’s compression modulus, elastic modulus, and fracture behavior [33]. This review’s findings highlight the potential of cocoon silk derivatives in advancing biomedical engineering and improving clinical outcomes.

## 5. Conclusions

This systematic review examined the mechanical properties and emerging clinical applications of different cocoon silks. The biocompatibility and strength of silk have profound implications for biomedical engineering, and our review has identified several applications ranging from tissue-engineering and energy-harvesting devices to antimicrobial hydrogels and drug-delivery systems, highlighting the broad potential of cocoon silk derivatives in the medical field. Despite the promising applications, several challenges remain.

Synthetic cocoon silk derivatives show significant promise in advancing biomedical engineering and improving clinical outcomes. These materials can play a crucial role in developing innovative medical devices and treatments that enhance patient care and outcomes by addressing the need for a standardized approach to transparently reporting mechanical properties and the identified complications and further exploring their applications. The continuing investigation into cocoon silk derivatives is essential for fully realizing their potential and bringing their benefits closer to the forefront of medical practice.

## Data Availability

The original contributions presented in the study are included in the article; further inquiries can be directed to the corresponding author.

## Supplementary Materials

The following supporting information can be downloaded at https://www.mdpi.com/article/10.3390/biomimetics9110675/s1. Table S1: NIH Quality Assessment Tool.
