# Supplementary material for: Mechanical Properties of Cocoon Silk Derivatives for Biomedical Application: A Systematic Review
Source: Biomimetics (Basel). 2024 Nov 6;9(11):675. doi: 10.3390/biomimetics9110675 (PMC11592187; doi:10.3390/biomimetics9110675)
Supplement: Supplementary file 1 [file biomimetics-09-00675-s001.zip › biomimetics-3186521-supplementary.pdf]

Supplemental Digital Content 1, Table S1. NIH quality assessment tool

**Article**	**Score**
A multi-layered nerve guidance conduit design adapted to facilitate surgical implantation.	Good
Biocompatible Silk/Polymer Energy Harvesters Using Stretched Poly (vinylidene fluoride-co-hexafluoropropylene) (PVDF-HFP) Nanofibers.	Fair
Characterization of direct ink write pure silk fibroin based on alcohol post-treatments	Good
Cross-linking of dialdehyde carboxymethyl cellulose with silk sericin to reinforce sericin film for potential biomedical application.	Good
Modification of sericin-free silk fibers for ligament tissue engineering application.	Good
Polyvinylidene fluoride/silk fibroin-based bio-piezoelectric nanofibrous scaffolds for biomedical application.	Good
Preparation and Characterization of a Silk Fibroin/Polyurethane Fiber Blend Membrane Containing Actinomycin X2 with Excellent Mechanical Properties and Enhanced Antibacterial Activities.	Good
Silk and Silk Composite Aerogel-Based Biocompatible Triboelectric Nanogenerators for Efficient Energy Harvesting	Good
Superb Silk Hydrogels with High Adaptability, Bioactivity, and Versatility Enabled by Photo-Cross-Linking.	Good
